# Targeting senescence-associated secretory phenotype macrophage: apigenin inhibits DOT1L-dependent H3K79me2 at *IL1A* to fight SASP in senescent macrophages

**DOI:** 10.3389/fmolb.2026.1801780

**Published:** 2026-07-16

**Authors:** Guozhen Ni, Ning Ma, Jian Xu

**Affiliations:** 1 Department of Cardiovascular Medicine, The First People’s Hospital of Linping District, Hangzhou, Hangzhou, Zhejiang, China; 2 Department of Cardiovascular Medicine, Heze Municipal Hospital, Shandong Provincial Hospital, Heze, Shandong, China

**Keywords:** apigenin, atherosclerosis, DOT1-like histone lysine methyltransferase, macrophages, senescence-associated secretory phenotype

## Abstract

**Introduction:**

Apigenin (API) has been utilized in the treatment of atherosclerosis. Senescent macrophages exhibit a senescence-associated secretory phenotype (SASP), which plays a critical role in the progression of atherosclerosis. *IL1A* expression is thought to be involved in the transition of macrophages to the SASP phenotype. This study explored the effect of API on the atherosclerosis-related SASP in macrophages and the underlying mechanism.

**Methods:**

Macrophages were treated with different concentrations of API. The expression of DOT1-like histone lysine methyltransferase (DOT1L) was manipulated in macrophages. Stimulation with lipopolysaccharide (LPS) was conducted to induce the SASP. Macrophage senescence was detected by senescence-associated-β-galactosidase staining. The expressions of interleukin 1 α (*IL1A*), DOT1L, and markers related to senescence, the SASP, and inflammation in macrophages were assessed. Chromatin immunoprecipitation was performed to check DOT1L-mediated H3K79me2 enrichment at the promoter of *IL1A*.

**Results:**

API treatment effectively reduced cellular senescence in LPS-exposed macrophages. Furthermore, it downregulated markers associated with senescence, SASP, and inflammation, while upregulating the anti-inflammatory cytokine IL-10. Moreover, LPS-induced DOT1L and H3K79me2 enrichment at the *IL1A* promoter, as well as DOT1L and *IL1A* upregulation in macrophages, were hindered by API. DOT1L overexpression counteracted API’s effects, including its ability to inhibit senescence, downregulate senescence/SASP/inflammation markers, upregulate IL-10, and prevent H3K79me2 and DOT1L binding at the *IL1A* promoter. DOT1L silencing reduced LPS-induced senescence, SASPs, and H3K79me2 enrichment in macrophages.

**Conclusion:**

API inhibits DOT1L-mediated H3K79me2 enrichment to downregulate *IL1A*, thus reducing the SASP of macrophages.

## 1 Introduction

Atherosclerosis is a chronic inflammatory vascular disease that stems from the formation of plaques and remains the leading cause of morbidity and mortality in the elderly (Zhu et al., 2018; Benjamin et al., 2019). Atherosclerotic plaques arise from the deposition of several plasma lipoproteins in the intima (Wang et al., 2017). One of the retained lipoproteins, low-density lipoprotein (LDL), is enzymatically/oxidatively modified and then activates endothelial cells by inducing their surface expression of adhesion molecules that elicit immune cell infiltration (Pirillo et al., 2013). Macrophages, which are differentiated from the infiltrating cells, take up lipoproteins, generating lipid-laden “foam cells,” the early hallmark of atherosclerotic plaques (Libby et al., 2019).

Macrophages are a key innate immune component, and their interaction with modified lipoproteins triggers proinflammatory cascades to boost the production of inflammatory cytokines/chemokines and reactive oxygen/nitrogen species within the growing lesions, thereby maintaining the local inflammatory response (Barrett, 2020). This persistent inflammation in turn elicits apoptosis and defective efferocytosis, contributing to debris accumulation, which facilitates necrotic core formation to render atherosclerotic plaque destabilization (Bäck et al., 2019; Barrett, 2020; Farahi et al., 2021), a high-risk factor for acute cardiovascular diseases (Kobiyama and Ley, 2018). Conversely, enhanced efferocytosis exerted by normal macrophages is a mechanism for reducing atherosclerosis progression by resolving plaque inflammation and debris (Thorp et al., 2011; Barrett, 2020).

The progressive formation of atherosclerotic lesions is known as an age-dependent process (Anagnostis et al., 2009). The inflammation propagation by macrophages has been proven to intensify with aging (Qian et al., 2012), while the macrophage-mediated phagocytic effect of apoptotic cells weakens under the aging condition (Boyd et al., 2012). Senescent macrophages exist in atherosclerosis, where macrophages undergo morphological alteration to present a senescence-associated secretory phenotype (SASP), which features the secretion of signaling molecules, including anti-proliferative proteins, proinflammatory mediators, and extracellular matrix-degrading enzymes (Kumari and Jat, 2021). The SASP correlates with the progression of atherosclerosis-associated inflammation and plaque formation (Childs et al., 2016; Wang et al., 2020). The findings above highlight the significance of targeting macrophage senescence in the treatment of atherosclerosis.

Interleukin 1 α (*IL1A*) is a proinflammatory cytokine from the interleukin 1 family (Apte and Voronov, 2008). SASPs of highly proinflammatory monocytes can be stimulated by *IL1A* (Ong et al., 2018), and upregulation of *IL1A* is linked to vascular inflammation in atherosclerosis (van den Hoek et al., 2022), which indicates that *IL1A* expression may be pertinent to the macrophage phenotype conversion into the SASP. *IL1A* is a proinflammatory factor that promotes plaque formation and inflammatory response in atherosclerosis. IL-10 is an anti-inflammatory factor that has a protective effect, suppressing inflammation and stabilizing plaque. Histone modification is an epigenetic control of all DNA-based processes, including transcription (Lawrence et al., 2016). Methylation occurs at the ε-amino group of histone lysine (Murray, 1964) and succumbs to the tight regulation of lysine methyltransferases and demethylases (Black et al., 2012). DOT1-like histone lysine methyltransferase (DOT1L) is the unique methyltransferase for H3K79. Its methylation product, H3K79me2, enables DOT1L occupancy at the *IL1A* gene locus, leading to transcriptional activation of *IL1A* and subsequently driving the expression of SASP genes (Leon et al., 2021). Accordingly, hindering DOT1L-mediated H3K79me2 enrichment-induced *IL1A* activation may reduce the SASP of macrophages in atherosclerosis.

Apigenin (API), one of the flavonoids present principally as glycosides in many types of plants (Hostetler et al., 2017), has exhibited diverse functional activities covering anticancer (Rahmani et al., 2022), anti-hyperglycemic (Villa-Rodriguez et al., 2018), antioxidant (Fidelis et al., 2019), and anti-inflammatory (Lim et al., 2013), as well as type-specific anti-apoptotic effects (Zhou et al., 2018). Owing to its anti-inflammatory and apoptosis-modulating properties, API has been implicated in the prevention and therapy of chronic diseases like atherogenesis (Ren et al., 2018) and age-related diseases (Gupta et al., 2018). Previously, its role against atherosclerosis has been documented to involve inhibiting endothelial cell apoptosis (Duan et al., 2021), inducing apoptosis of lipid-laden macrophages, and downregulating the secretion of proinflammatory cytokines (Wang et al., 2015). Of note, prediction based on HERB indicates that the ingredients targeting *IL1A* include API, and API decreases the activity of histone methyltransferases to inhibit histone methylation (Kanwal et al., 2016). Leon et al. (2021) revealed the regulation of *IL1A* by DoT1L/H3K79me2, but its role in aging-related disease models (e.g., atherosclerosis) has not been clarified. This study aims to fill this gap and to explore whether API inhibits inflammation in atherosclerosis through this pathway.

Taken together, we hypothesized that API inhibits macrophages presenting a SASP in atherosclerosis by inhibiting DOT1L-mediated H3K79me2 enrichment at the *IL1A* gene. In this study, SASPs were induced in macrophages pretreated with API to verify the reliability of our hypothesis.

## 2 Methods and materials

### 2.1 Cell culture and induction

Human THP-1 monocytes were purchased from COBIOER Bioscience (CBP60518, Nanjing, China) and maintained in their complete culture medium (CBP60518M, COBIOER, China) in a humidified incubator with 5% CO_2_ at 37 °C. THP-1 cells were adjusted to a suspension at a density of 1.0 × 10^5^ cells/mL and then induced into macrophages by being stimulated with 100 ng/mL 4β-phorbol-12-myristate-13-acetate (PMA) (P8139, pre-dissolved in ethanol, Sigma-Aldrich, St. Louis, MO, United States) for 24 h. After PMA stimulation for 24 h, the cells were washed twice with phosphate-buffered saline (PBS) and cultured in fresh complete medium without PMA for an additional 24 h to allow recovery and minimize residual activation before subsequent treatments.

### 2.2 Cell counting kit-8 (CCK-8) assay

A CCK-8 assay was performed to examine the cytotoxicity of API toward macrophages. Macrophages were seeded in 96-well plates at a density of 2 × 10^3^ cells/well and cultured until they adhered to the plate, followed by treatment with API (SMB00702, C_15_H_10_O_5_, purity: ≥97%, pre-dissolved in dimethyl sulfoxide, Sigma-Aldrich, United States) at 0, 10, 20 and 50 µM for 0, 6, 12 and 24 h (Wang et al., 2015). Later, 10 µL of CCK-8 reagent (CA1210, Solarbio, Beijing, China) was added per well and incubated with the cells for 2 h at 37 °C. A microplate reader (EMax Plus, Molecular Devices, Sunnyvale, CA, United States) was used to read the optical density at 450 nm. Cell viability was determined as follows: Cell viability (%) = (OD_Experimental_ − OD_blank_)/(OD_Control_ − OD_blank_) × 100.

### 2.3 Cell transfection

Overexpression plasmids for DOT1L (RC218547, OriGene, Rockville, MD, United States), a negative control for the overexpression plasmids (NC; PS100001, OriGene, United States), DOT1L small interfering RNA (siRNA) and negative control (NC) siRNA (GenePharma, Shanghai, China) were separately transfected into macrophages with the help of Lipofectamine 3,000 transfection reagent (L3000015, ThermoFisher, Waltham, MA, United States). Briefly, macrophages were seeded in 96-well plates (1 × 10^4^ cells/well) and cultured until an 80% cell confluency. After being diluted with Opti-MEM Medium (10 μL) and P3000 Reagent (0.4 μL), the above plasmids (0.2 μg) or siRNA and Lipofectamine 3000 transfection reagent (0.45 μL) were incubated together for 10 min at 37 °C to obtain gene-lipid complexes. The cells were incubated with the gene–lipid complexes was conducted for 48 h with the cells and were then subjected to the assessment of transfection efficiency by quantitative reverse transcription-polymerase chain reaction (qRT-PCR).

### 2.4 API treatment and SASP induction

In experiments other than the CCK-8 assay, transfected/non-transfected macrophages were treated with 10 and 20 µM API for 12 h in the culture medium (Wang et al., 2015), after which 1 μg/mL lipopolysaccharide (LPS; L2630, Sigma-Aldrich, United States) was added to stimulate the macrophages for 24 h to induce cellular senescence (Wang et al., 2020).

### 2.5 Western blot

Whole-cell proteins (4 × 10^5^ cells/mL) were harvested using RIPA Lysis Buffer (20–188, Sigma-Aldrich, United States) supplemented with a protease inhibitor (P8340, Sigma-Aldrich, United States) from non-transfected macrophages that had undergone API treatment and LPS stimulation. A BCA kit (A53227, ThermoFisher, United States) was utilized to determine the protein concentration. Then, the proteins were separated via electrophoresis on a sodium dodecyl sulfate polyacrylamide gel electrophoresis (SDS-PAGE) gel (1615100, BIO-RAD, Hercules, CA, United States), and transferred onto a polyvinylidene fluoride membrane (1620256, BIO-RAD, United States). The membrane was blocked in 5% skimmed milk for 1 h at room temperature, followed by incubation with primary antibodies for *IL1A* (ab300501, 1:1000, Abcam, United Kingdom), DOT1L (ab72454, 2 μg/mL, Abcam, United Kingdom), and glyceraldehyde-3-phosphate dehydrogenase (GAPDH) (ab8245, 1:500, Abcam, United Kingdom) overnight at 4 °C. The membrane was washed with TBS-T buffer (28360, ThermoFisher, United States) and probed with goat anti-rabbit/mouse IgG secondary antibodies (ab97051/ab6789, Abcam, United Kingdom). Immunoreactive bands were visualized on a luminescence imager (ChemiDoc, BIO-RAD, United States) using ECL Substrate (32109, ThermoFisher, United States), and analyzed using ImageJ software (3.0 version, National Institutes of Health, Bethesda, MA, United States). The normalized data were compared with the control group to calculate the relative expression multiple. Relative expression multiple = The relative expression level was normalized in the experimental group/The relative expression level of the control group was normalized. All Western blot experiments were performed with three independent biological replicates (n = 3).

### 2.6 Senescence-associated-β-galactosidase (SA-β-gal) staining

SA-β-Gal staining was performed using a senescence β-Galactosidase Staining Kit (C0602, Beyotime, Shanghai, China). The macrophages were seeded in 6-well plates (1 × 10^6^ cells/well) and fixed with 4% paraformaldehyde (P0099, Beyotime, China) for 15 min at room temperature. After being rinsed with phosphate-buffered saline (PBS; P4417, Sigma-Aldrich, United States) thrice, the cells were incubated overnight at 37 °C with 1 mL of SA-β-gal staining working solution, protected from CO_2_.

Later, SA-β-gal-positive macrophages were detected by using an optical microscope (ECLIPSE TS100, Nikon, Tokyo, Japan) under a ×200 magnification. ImageJ software randomly counts blue cells in multiple fields (SA-β-gal positive). Relative SA-β-gal positive cells = The number of positive cells in the experimental group/The number of positive cells in the control group.

### 2.7 Chromatin immunoprecipitation (CHIP)-quantitative polymerase chain reaction (qPCR)

The macrophages (1 × 10^6^) were fixed in 1% formaldehyde for 10 min for protein-DNA crosslinking, which was later quenched using 2.5 M glycine. The cells were then lysed using RIPA Lysis Buffer and subjected to ultrasonication for shearing chromatin into fragments ranging from 300 to 700 bp. Centrifugation at 10000× g for 10 min was performed at 4 °C to obtain the supernatant. The supernatant was diluted and pre-cleared with 20 μL of 50% protein A-agarose beads (21348, ThermoFisher, United States) for 3 h. An antibody for H3K79me2 (ab177184, Abcam, United Kingdom) or DOT1L (ab72454, Abcam, Cambridge, United Kingdom) was used to immunoprecipitate chromatin in the supernatant overnight at 4 °C. Normal rabbit IgG (ab171870, Abcam, United Kingdom) was added as the NC group. The immune complexes were then incubated with 40 μL 50% protein A-agarose beads at 4 °C for 2 h. Thereafter, the beads were rinsed with CHIP buffer (20 mM Tris, pH 7.9, 2 mM EDTA, 0.05% SDS, 250 mM NaCl, and 0.25% Triton X-100), resuspended in 10 µL of elution buffer, and de-crosslinked at 65 °C overnight, followed by protein digestion with 0.05 mg/mL Proteinase K (P274341, Aladdin, Shanghai, China) at 37 °C for 1 h. Finally, the promoter region of *IL1A* was detected through qPCR. Quantification of CHIP enrichment was performed using the percent input method. Briefly, the threshold cycle (Ct) values were obtained for the input chromatin (diluted 1:100) and for the immunoprecipitated (IP) samples. The percent input for each sample was calculated as: % Input = 2^−ΔCt^ × 100%, ΔCt = Ct (IP) − Ct (Input, corrected), Ct (Input, corrected) = Ct (Input) − log2(Input dilution factor). Enrichment values were then normalized to the NC (normal rabbit IgG) and expressed as fold change relative to the control group.

### 2.8 QRT-PCR

Total RNA was extracted from the macrophages (4 × 10^5^ cells/mL) with RNeasy Mini kits (74106, Qiagen, Düsseldorf, Germany), and quantified using a spectrophotometer (NanoDrop 2000, ThermoFisher, United States). The RNA was used as a template for synthesizing cDNA using reverse transcription kits (K1622, Yaanda Biotechnology, Beijing, China). qPCR was implemented with a detection system (LightCycler 96, Roche, Indianapolis, IN, United States) using Eastep qPCR Master Mix (LS 2062, Promega, Madison, WI, United States). The following amplification conditions were used: 95 °C for 10 min and 40 cycles of 95 °C for 15 s and 60 °C for 1 min. Predesigned primers are listed in Table 1. Relative mRNA expressions were determined using the 2^−ΔΔCt^ method (Livak and Schmittgen, 2001), while being normalized to GAPDH.

**TABLE 1:** Primers used in quantitative reverse transcription-polymerase chain reaction/chromatin immunoprecipitation-quantitative polymerase chain reaction for related genes.

Genes	Species	Forward (5’- -3′)	Reverse (5’- -3′)
*P21*	Human	TGTCCGTCAGAACCCATGC	AAAGTCGAAGTTCCATCGCTC
*P16*	Human	GATCCAGGTGGGTAGAAGGTC	CCCCTGCAAACTTCGTCCT
*IL-6*	Human	ACTCACCTCTTCAGAACGAATTG	CCATCTTTGGAAGGTTCAGGTTG
*VEGFC*	Human	GAGGAGCAGTTACGGTCTGTG	TCCTTTCCTTAGCTGACACTTGT
*TNF-α*	Human	CCTCTCTCTAATCAGCCCTCTG	GAGGACCTGGGAGTAGATGAG
*IL-1β*	Human	ATGATGGCTTATTACAGTGGCAA	GTCGGAGATTCGTAGCTGGA
*CXCL10*	Human	GTGGCATTCAAGGAGTACCTC	TGATGGCCTTCGATTCTGGATT
*IL-10*	Human	GACTTTAAGGGTTACCTGGGTTG	TCACATGCGCCTTGATGTCTG
*DOT1L*	Human	CGCTGCCGGTCTACGATAAA	TCGATGGCACGGTTGTACTT
*IL1A*	Human	ATGTGCATTGGCTTCTCCCA	ACATCCTGATGAAGCCTGCC
*GAPDH*	Human	GGAGCGAGATCCCTCCAAAAT	GGCTGTTGTCATACTTCTCATGG

### 2.9 DOT1L enzymatic activity assay

The catalytic activity of DOT1L was assessed utilizing a commercially available assay system. Recombinant human DOT1L protein (25 ng/μL; BPS Bioscience, Cat. No. 52202; San Diego, CA, United States) was incubated with a synthetic peptide substrate in the presence of S-adenosyl-L-methionine (SAM, 5 µM), which served as the methyl donor. All reactions were performed in strict accordance with the protocols provided by the manufacturer.

### 2.10 Statistical analysis

All results are presented as the mean ± standard deviation (SD) of experiments conducted in triplicate with three independent biological replicates. Statistical analysis was carried out using GraphPad Prism (version 8.0, GraphPad Software Inc., San Diego, CA, United States). One-way analysis of variance (ANOVA) followed by Tukey’s *post hoc* test was used for comparisons among multiple groups. Statistical significance was accepted at *P <* 0.05.

## 3 Results

### 3.1 Treatment with API at 10 and 20 µM for 12 h delivered no impact on macrophage viability while resisting senescence of LPS-exposed macrophages

**FIGURE 1:**
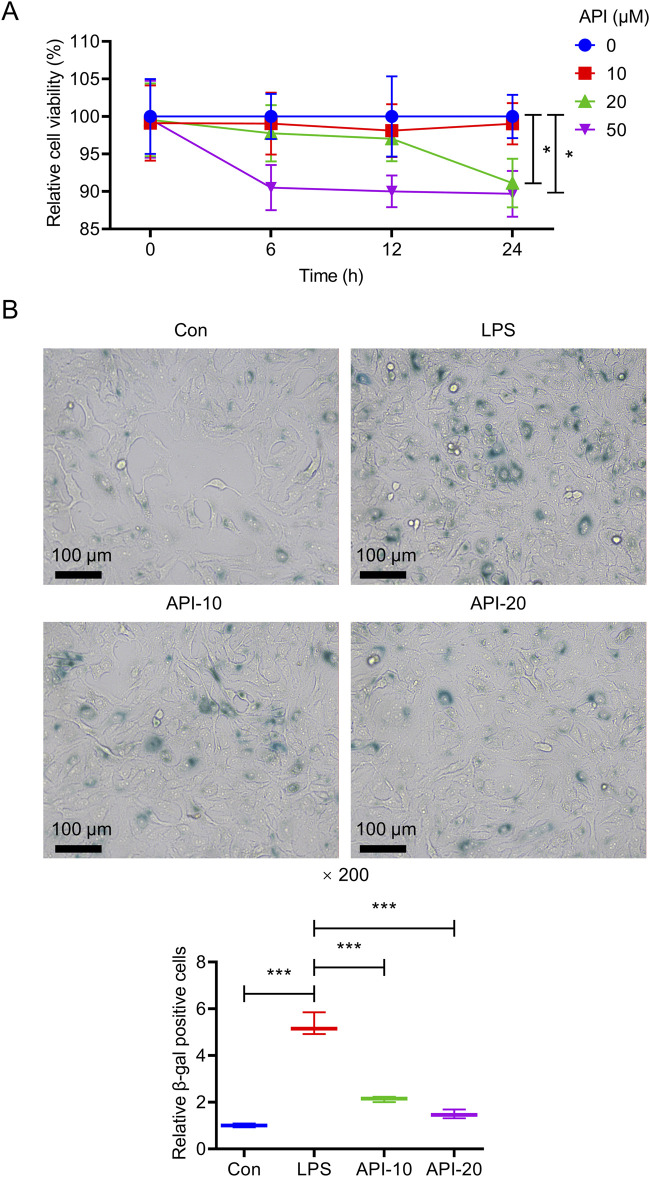
Treatment with API at 10 and 20 µM for 12 h delivered no impact on macrophage viability while resisting senescence of LPS-exposed macrophages. **(A)** The viability of THP-1 macrophages treated with 0, 10, 20, and 50 µM API for 0, 6, 12, and 24 h was determined by cell counting kit-8 assay. **(B)** Senescence of THP-1 macrophages that had been treated with 10 and 20 µM API for 12 h and stimulated with 1 μg/mL LPS for 24 h was detected by senescence-associated-β-galactosidase staining (magnification, ×200; scale bar, 100 µm). ^*^ *P* < 0.05, ^***^ *P* < 0.001. n = 3 per group. (API, apigenin; Con, control; LPS, lipopolysaccharide).

A CCK-8 assay and SA-β-gal staining were performed to detect the effect of API on macrophage viability and aging. The data from the CCK-8 assay detected no obvious changes in the viability of macrophages under treatment with API (10 µM) (Figure 1A), while showing that API (20 µM) treatment reduced the viability of macrophages at 24 h post-treatment and the viability also declined in macrophages after 6, 12 and 24 h of API (50 µM) treatment (Figure 1A, *P* < 0.05), which may be because higher concentrations of API may cause cell death through induction of apoptosis or oxidative stress, which is also similar to that reported in a previous study (Hu et al., 2019). Therefore, API (10 and 20 µM) was selected to treat macrophages for 12 h in the subsequent experiments. Macrophages were induced by LPS to exhibit a SASP. LPS stimulation incurred macrophage senescence with an increased number of cells positive for SA-β-gal (Figure 1B, *P* < 0.001), which, however, was mitigated by API (10 and 20 µM) treatment (Figure 1B, *P* < 0.001).

### 3.2 API treatment reduced LPS-induced senescence, SASPs, and inflammation in macrophages

**FIGURE 2:**
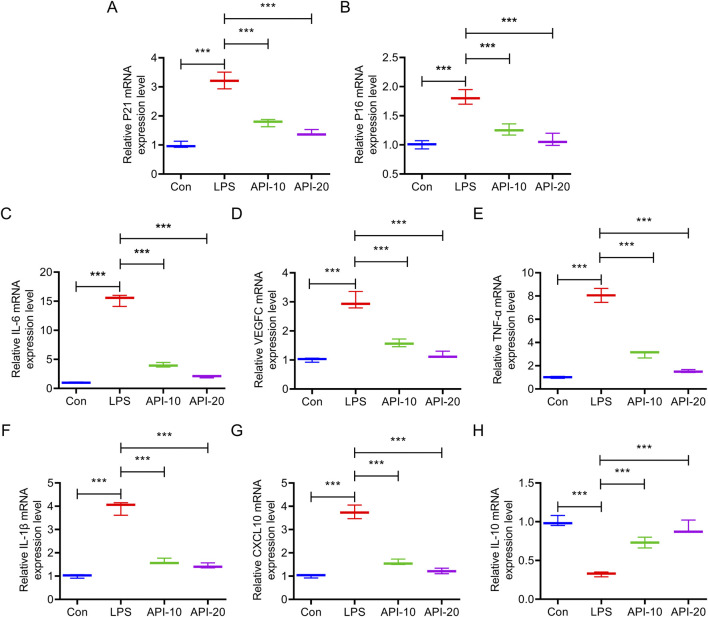
API treatment reduced LPS-induced senescence, SASP, and inflammation in macrophages. **(A–H)** The expressions of markers related to senescence (P21 and P16), the SASP (IL-6, VEGFC, and TNF-α), and inflammation (IL-1β,CXCL10, and IL-10) in THP-1 macrophages that had been treated with 10 and 20 µM API for 12 h and stimulated with 1 μg/mL LPS for 24 h were assessed by qRT-PCR, with GAPDH serving as the normalizer. ^***^ *P* < 0.001. n = 3 per group. (API, apigenin; Con, control; LPS, lipopolysaccharide; IL-6, interleukin-6, VEGFC, vascular endothelial growth factor C; TNF-α; tumor necrosis factor-α; IL-1β, interleukin-1β; CXCL10, C–X–C motif chemokine 10; IL-10; interleukin-10; qRT-PCR, quantitative reverse transcription-polymerase chain reaction; SASP, senescence-associated secretory phenotype; GAPDH, glyceraldehyde-3-phosphate dehydrogenase).

To explore the effects of API on LPS-induced macrophage senescence, SASPs, and inflammation, qRT-PCR experiments were performed in this section. According to qRT-PCR analysis, API (10 and 20 µM) treatment resisted LPS-induced upregulation of P21 and P16 in macrophages (Figures 2A,B, *P* < 0.001). API (10 and 20 µM) treatment decreased the levels of SASP-related markers, IL-6, VEGFC, and TNF-α in LPS-induced macrophages (Figures 2C–E, *P* < 0.001). Enhanced mRNA expression of IL-1β and CXCL10, as well as reduced IL-10 expression was detected in macrophages upon LPS stimulation (Figures 2F–H, *P* < 0.001). Macrophages treated with API (10 and 20 µM) and LPS had lower levels of IL-1β and CXCL10 and a higher IL-10 level, when compared to macrophages that only experienced LPS stimulation (Figures 2F–H, *P* < 0.001).

### 3.3 API treatment hindered DOT1L-mediated H3K79me2 enrichment to downregulate *IL1A* in LPS-induced macrophages

**FIGURE 3:**
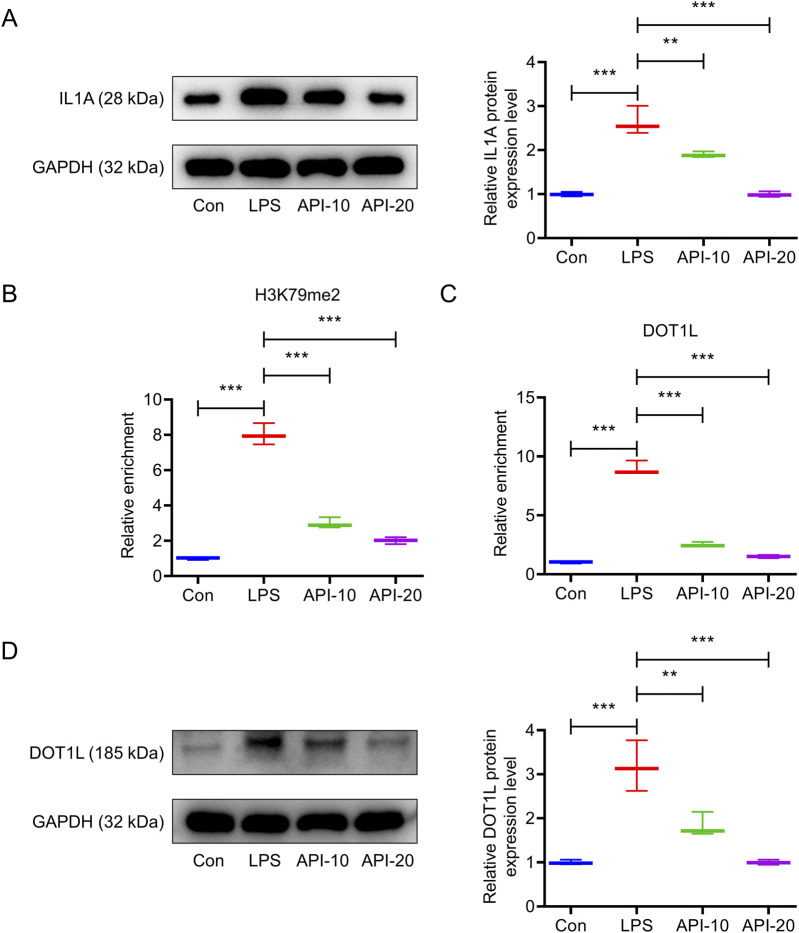
API treatment hindered DOT1L-mediated H3K79me2 enrichment to downregulate *IL1A* in LPS-induced macrophages. **(A–D)** THP-1 macrophages were treated with 10 and 20 µM API for 12 h and stimulated with 1 μg/mL LPS for 24 h **(A)**. The expression of *IL1A* in THP-1 macrophages was assessed by Western blot, with GAPDH serving as the normalizer. **(B,C)** DOT1L-mediated H3K79me2 enrichment at the promoter of *IL1A* in THP-1 macrophages was checked by CHIP-qPCR. **(D)** The expression of DOT1L in THP-1 macrophages was assessed by Western blot, with GAPDH serving as the normalizer. ^**^ *P* < 0.01, ^***^ *P* < 0.001. n = 3 per group. (API, apigenin; Con, control group; LPS, lipopolysaccharide; *IL1A*, interleukin-1α; CHIP-qPCR, chromatin immunoprecipitation-quantitative polymerase chain reaction; DOT1L, DOT1-like histone lysine methyltransferase; GAPDH, glyceraldehyde-3-phosphate dehydrogenase).

In this part, WB and CHIP-qPCR were used to investigate the regulation of DOT1L and *IL1A* by API treatment and to explore the H3K79me2 and DOT1L enrichment in the *IL1A* promoter region. Exposure to LPS-induced *IL1A* expression in macrophages (Figure 3A, *P* < 0.001), which was neutralized by API (10 and 20 µM) treatment (Figure 3A, *P* < 0.01). Through CHIP-qPCR, the H3K79me2 and the unique writer of H3K79 methylation (DOT1L), were found to be enriched in the promoter region of *IL1A* in macrophages following LPS stimulation (Figures 3B,C, *P* < 0.001); however, API (10 and 20 µM) treatment could lessen this enrichment of H3K79me2 and DOT1L (Figures 3B,C, *P* < 0.001). LPS stimulation in macrophages caused an increase in DOT1L expression (Figure 3D, *P* < 0.001), but this increase in DOT1L expression was attenuated by API (10 and 20 µM) treatment (Figure 3D, *P* < 0.01). Since more obvious changes were detected in LPS-induced macrophages treated with 20 µM API than in those treated with 10 µM API, 20 µM was the therapeutic dose of API used in the subsequent experiments.

### 3.4 DOT1L overexpression impaired API-imposed resistance to senescence in LPS-exposed macrophages

**FIGURE 4:**
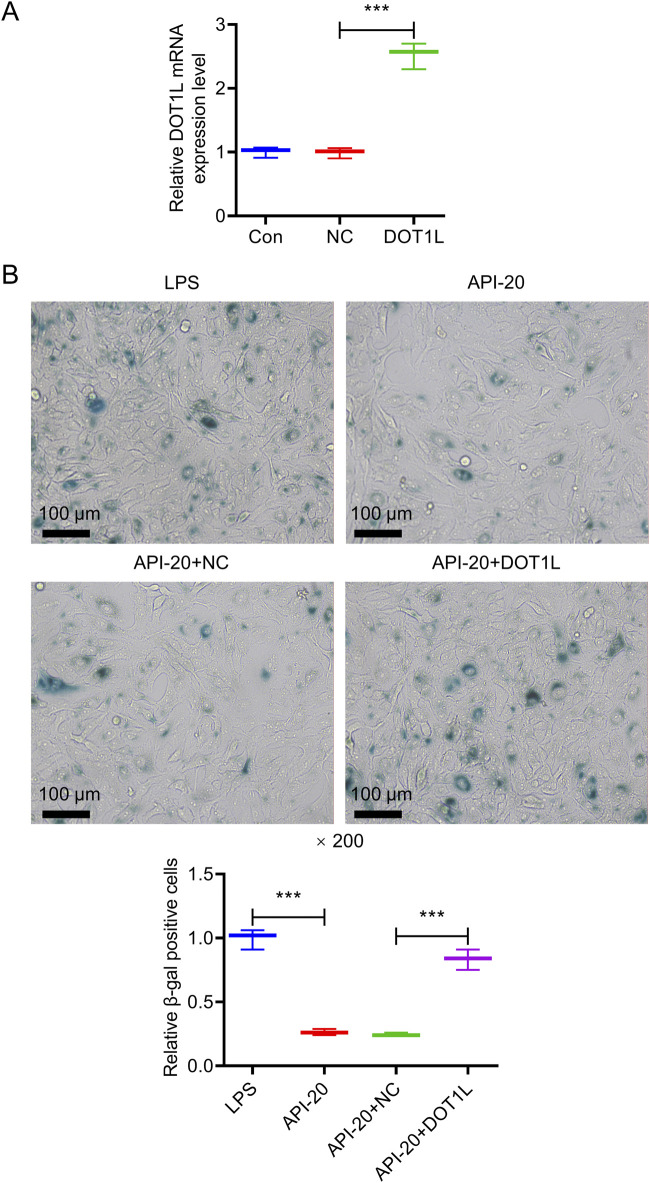
DOT1L overexpression impaired API-imposed resistance to senescence in LPS-exposed macrophages. **(A)** The expression of DOT1L in THP-1 macrophages transfected with NC/DOT1L overexpression plasmids was assessed by qRT-PCR, with GAPDH serving as the normalizer. **(B)** Senescence of THP-1 macrophages that had undergone transfection with NC/DOT1L overexpression plasmids and treatment with 20 µM API for 12 h, followed by stimulation with 1 μg/mL LPS for 24 h was detected by senescence-associated-β-galactosidase staining (magnification, ×200; scale bar, 100 µm). ^***^ *P* < 0.001. n = 3 per group. (API, apigenin; LPS, lipopolysaccharide; NC, negative control; DOT1L, DOT1 like histone lysine methyltransferase; GAPDH, glyceraldehyde-3-phosphate dehydrogenase).

SA-β-gal staining was performed to detect the effect of API on macrophage senescence. Transfection with DOT1L overexpression plasmids induced Dan increase in DOT1L expression in macrophages (Figure 4A, *P* < 0.001). API (20 µM) treatment delivered resistance to LPS-induced macrophages senescence, which, however, was abolished by DOT1L overexpression (Figure 4B, *P* < 0.001).

### 3.5 DOT1L overexpression abrogated API-delivered reduction on SASPs and H3K79me2 enrichment at the *IL1A* promoter in LPS-exposed macrophages

**FIGURE 5:**
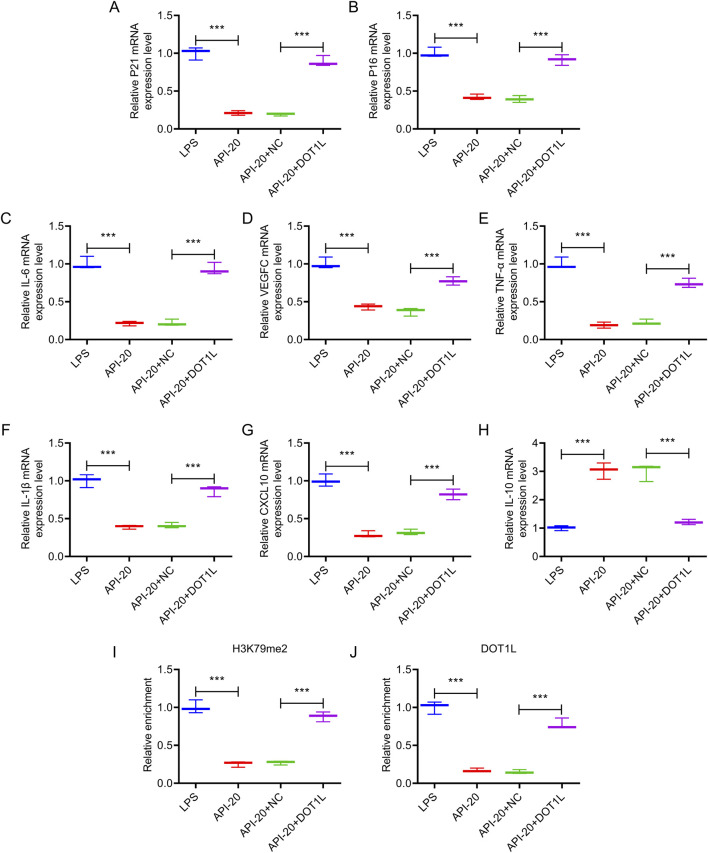
DOT1L overexpression abrogated API-delivered reduction in the SASPs and H3K79me2 enrichment at the *IL1A* promoter in LPS-exposed macrophages. **(A–J)** THP-1 macrophages were subjected to transfection with NC/DOT1L overexpression plasmids and treatment with 20 µM API for 12 h, followed by stimulation with 1 μg/mL LPS for 24 h. **(A–H)**. The expressions of markers related to senescence (P21 and P16), the SASP (IL-6, VEGFC, and TNF-α) and inflammation (IL-1β, CXCL10 and IL-10) in THP-1 macrophages were assessed by qRT-PCR, with GAPDH serving as the normalizer. **(I,J)** DOT1L-mediated H3K79me2 enrichment at the promoter of *IL1A* was checked by CHIP-qPCR. ^***^ *P* < 0.001. n = 3 per group. (API, apigenin; LPS, lipopolysaccharide; NC, negative control; IL-6, interleukin-6; VEGFC, vascular endothelial growth factor C; TNF-α, tumor necrosis factor-α; IL-1β, interleukin-1β; βCXCL10, C–X–C motif chemokine 10; IL-10, interleukin-10; qRT-PCR, quantitative reverse transcription-polymerase chain reaction; CHIP-qPCR, chromatin immunoprecipitation-quantitative polymerase chain reaction; GAPDH, glyceraldehyde-3-phosphate dehydrogenase; SASP, senescence-associated secretory phenotype).

**FIGURE 6:**
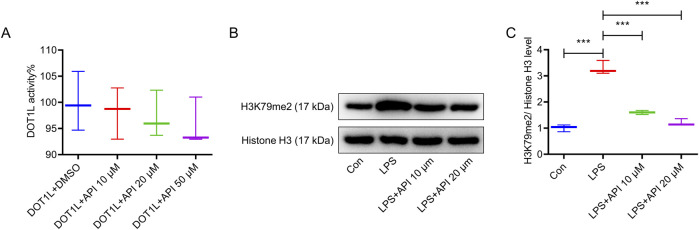
Effects of API on DOT1L enzyme activity and global H3K79me2 levels. **(A)** A DOT1L enzyme activity assay was analyzed after incubation with API (10, 20, and 50 µM). **(B,C)** The expression levels of H3K79me2 were determined by Western blotting. Histone H3 was used as an internal control. ^***^ *P* < 0.001. n = 3 per group.

In this part, qRT-PCR and CHIP-qPCR were used to investigate the regulation of DOT1L in the inflammatory response induced by macrophages by LPS and explore the H3K79me2 and DOT1L enrichment in the *IL1A* promoter region. The expressions of P21 and P16 in LPS-exposed macrophages were repressed by 20 µM API (Figures 5A,B, *P* < 0.001), whereas DOT1L overexpression revoked this repression in P21 and P16 expressions (Figures 5A,B, *P* < 0.001). Meanwhile, DOT1L overexpression abolished the 20 µM API-conferred inhibition of high IL-6, VEGFC, and TNF-α levels induced by LPS in macrophages (Figures 5C–E, *P* < 0.001). Besides, the 20 µM API-induced decline in IL-1β and CXCL10 expressions and upregulation of IL-10 expression in LPS-induced macrophages were withdrawn by DOT1L overexpression (Figures 5F–H, *P* < 0.001). Of note, DOT1L overexpression resisted the 20 µM API-imposed attenuation of LPS-elicited H3K79me2 and DOT1L enrichment at the *IL1A* promoter in macrophages (Figures 5I,J, *P* < 0.001). To investigate whether API directly inhibits the DOT1L enzymatic activity, we conducted an assay. The results showed that API had no significant effect on the DOT1L enzymatic activity (Figure 6A). In addition, the effect of API on the global H3K79 dimethylation level in macrophages was evaluated. The results showed that after API treatment, H3K79me2 level decreased in a concentration-dependent manner (Figures 6B,C, *P* < 0.001).

### 3.6 DOT1L silencing reduced LPS-induced senescence, SASPs and H3K79me2 enrichment in macrophages

**FIGURE 7:**
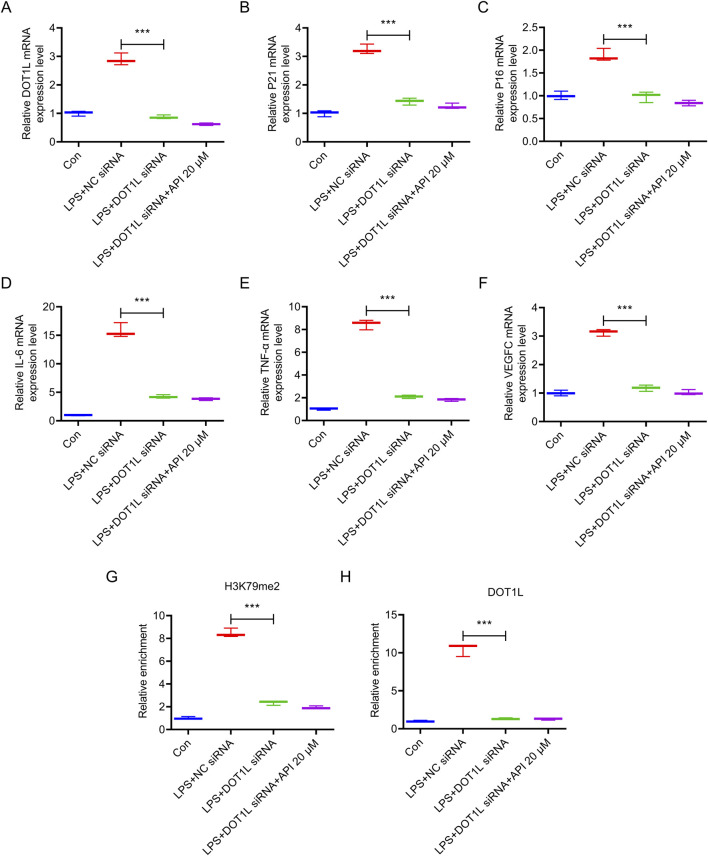
Effects of DOT1L silencing and API on the SASP and H3K79me2 enrichment at the *IL1A* promoter in LPS-exposed macrophages. **(A–F)** The expressions of DOT1L, markers related to senescence (p21 and p16), and the SASP (IL-6, VEGFC, and TNF-α) in THP-1 macrophages were assessed by qRT-PCR, with GAPDH serving as the normalizer. **(G,H)** DOT1L-mediated H3K79me2 enrichment at the promoter of *IL1A* was assessed by CHIP-qPCR. ^***^ *P* < 0.001. n = 3 per group.

To explore the necessity of DOT1L for the SASP phenotype of macrophages, and whether the effect of API depends on DOT1L, senescence and SASPs were detected by qRT-PCR experiments. According to qRT-PCR analysis, DOT1L silencing inhibited the LPS-induced upregulation of DOT1L, P21, and P16 in macrophages (Figures 7A–C, *P* < 0.001). Compared with the LPS + DOT1L siRNA group, API treatment had no significant effect on these indicators (Figures 6A–C). DOT1L silencing decreased the levels of SASP-related markers, IL-6, VEGFC, and TNF-α in LPS-induced macrophages (Figures 7D–F, *P* < 0.001), and API treatment had no significant effect on these indicators compared with the LPS + DOT1L siRNA group (Figures 7D–F). In addition, DOT1L silencing resisted LPS-elicited H3K79me2 and DOT1L enrichment at the *IL1A* promoter in macrophages (Figures 7G,H, *P* < 0.001).

### 3.7 DOT1L overexpression enhanced the effect of LPS on the senescence, SASPs, and H3K79me2 enrichment in macrophages

**FIGURE 8:**
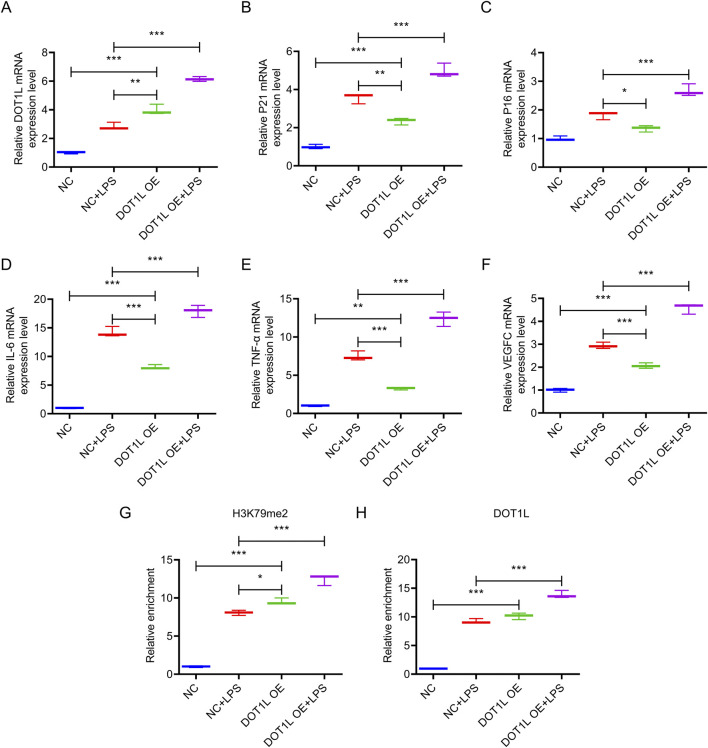
Effects of DOT1L overexpression on the SASP and H3K79me2 enrichment at the *IL1A* promoter in LPS-exposed macrophages. **(A–F)** The expressions of DOT1L, markers related to senescence (p21 and p16), and the SASP (IL-6, VEGFC, and TNF-α) in THP-1 macrophages were assessed by qRT-PCR, with GAPDH serving as the normalizer. **(G,H)** DOT1L-mediated H3K79me2 enrichment at the promoter of *IL1A* was assessed by CHIP-qPCR. ^*^ *P* < 0.05, ^**^ *P* < 0.01, ^***^ *P* < 0.001. n = 3 per group.

To investigate whether DOT1L overexpression alone (without LPS stimulation) is sufficient to induce the SASP, we conducted relevant research. The expression of DOT1L was increased after macrophages were transfected with a DOT1L overexpression plasmid or treated with LPS (Figure 8A, *P* < 0.01). DOT1L overexpression promoted the expression of P21, P16, IL-6, VEGFC, and TNF-α in macrophages (Figures 8B–F, *P* < 0.01), but the impact on these indicators was less than that caused by LPS stimulation alone. LPS stimulation further promoted the effect of DOT1L overexpression on these indicators (Figures 8B–F, *P* < 0.001). In addition, DOT1L overexpression increased the H3K79me2 and DOT1L enrichment at the *IL1A* promoter in macrophages (Figures 8G,H, *P* < 0.001), and LPS stimulation further promoted the H3K79me2 and DOT1L enrichment at the *IL1A* promoter in macrophages (Figures 8G,H, *P* < 0.001).

## 4 Discussion

Lipid accumulation in the artery intima induces endothelial cell activation, followed by the recruitment of immune cells, which are further involved in macrophage foam cell formation, an early marker of lipid-rich atherosclerotic plaques (Khoury et al., 2021). Foam macrophages undergoing senescence aggravate atherosclerotic lesions through promoting key atherogenic and inflammatory events (Childs et al., 2016). API has been previously identified as a foam cell-reducing and inflammation-resolving agent against atherosclerosis (Wang et al., 2015). The present study provided evidence to directly support that API also resists macrophage senescence in atherosclerosis and unveiled the underlying molecular mechanism.

The SASP is one hallmark of senescence, resulting in a proinflammatory microenvironment comprising a plethora of soluble signaling factors, such as inflammaging-related factors, matrix metalloproteinases, and adhesion molecules, when it accumulates (Coppé et al., 2008; Kuilman et al., 2008; Kumari and Jat, 2021). The SASPs of macrophages are induced in atherosclerosis, driving the expression of key atherogenic and inflammatory cytokines and also facilitating elastic fiber degradation and fibrous cap thinning to promote the instability of plaques (Childs et al., 2016; Wang et al., 2020). While LPS is primarily an acute inflammatory stimulus, accumulating evidence demonstrates that LPS exposure can induce senescence-like phenotypes in macrophages, characterized by SA-β-gal positivity, upregulation of p16/p21, and acquisition of a proinflammatory SASP profile (Fang et al., 2024; Wang et al., 2020). This model has been widely used to study inflammation-associated senescence (Suzuki et al., 2022). We acknowledge that classical senescence inducers such as Ox-LDL, Ang II, or replicative senescence may better recapitulate the chronic context of atherosclerosis. Future studies comparing multiple senescence models will be valuable.

API has been reported to suppress the SASP of breast cancer cells (Perrott et al., 2017) and bleomycin-treated fibroblasts (Lim et al., 2015). Moreover, treatment with API ameliorates atherosclerosis through resolving inflammation in ApoE (−/−) mice (Wang et al., 2015). The literature led us to hypothesize a guess that API reduces the SASP of macrophages. Cellular senescence represents an irreversible proliferative arrest involving extensive changes in the expression and secretion of proteins, which are mainly involved in the p53-p21 and p16-retinoblastoma protein pathways (Coppé et al., 2010; Lanigan et al., 2011). Among these, p21, a cyclin-dependent kinase (CDK) inhibitor protein that suppresses DNA synthesis and induces cell cycle arrest (Al Bitar and Gali-Muhtasib, 2019), is activated mainly at the early stage of senescence evolution (Dulić et al., 2000), while P16, another CDK inhibitory protein that negatively regulates cell proliferation (García-Reyes et al., 2018), maintains the senescence state (Dulić et al., 2000). The SA-β-gal positive level has high sensitivity and specificity for cellular senescence (Itahana et al., 2007). They were found to be downregulated in LPS-induced senescent macrophages with API treatment in our study. Meanwhile, the expressions of proinflammatory cytokines, interleukin (IL)-6 and tumor necrosis factor (TNF)-α as well as vascular endothelial growth factor C (VEGFC), all of which are markers of the SASP profile (Wang et al., 2020), declined upon API treatment in LPS-induced senescent macrophages. In addition to these, API-treated senescent macrophages showed lower expression levels of the proinflammatory cytokine, IL-1β, and the chemokine, C–X–C motif chemokine 10 (CXCL10), while showing higher expression levels of the anti-inflammatory cytokine, IL-10, than those without API treatment. The above results confirm that API hinders the conversion of the macrophage phenotype into the SASP.

The SASP-related proinflammatory cytokines and chemokines are transcriptionally upregulated by nuclear factor-κB, which is activated following the activation of its upstream gene, *IL1A* (Orjalo et al., 2009), making *IL1A* the main inducer of the SASP. High levels of *IL1A* also correlate with inflammation in atherosclerosis (van den Hoek et al., 2022). API has been previously proven to suppress *IL1A* expression in preclinical mouse models of Down syndrome (Guedj et al., 2020). Similarly, repressed *IL1A* expression was noted in senescent macrophages after API treatment, suggesting that API downregulates *IL1A* to reduce the SASP of macrophages. Histone methylation is an epigenetic modification that directly regulates transcription to affect gene expression (Lawrence et al., 2016). The histone marker H3K79 has been established as a regulator of senescence (Kim et al., 2012), and is associated with active transcription (Wood et al., 2018). We acknowledge that H3K79me2 is primarily associated with transcriptional elongation rather than promoter activation. However, emerging evidence suggests that DOT1L-mediated H3K79me2 enrichment at promoter regions can facilitate transcriptional initiation by promoting chromatin accessibility and recruiting transcription factors. DOT1L, the sole methyltransferase for H3K79, causes H3K79me2 enrichment at the *IL1A* gene locus, leading to the transcription of *IL1A*, which subsequently induces the expression of SASP genes (Leon et al., 2021). Nevertheless, the precise mechanistic link between H3K79me2 enrichment at the *IL1A* promoter and its transcriptional activation requires further investigation. In our study, the occupancy of H3K79me2 at the promoter of *IL1A* and DOT1L upregulation were observed in senescent macrophages and impaired, along with *IL1A* downregulation, by API treatment. DOT1L deletion results in the repression of *IL1A*, as well as other SASP genes during oncogene-induced senescence (Olan and Narita, 2021), consistent with which DOT1L overexpression abolished API-delivered inhibition of the expressions/levels of the above markers related to senescence, the SAPS and inflammation, as well as on the inhibition of DOT1L-mediated H3K79me2 enrichment at the *IL1A* gene in senescent macrophages in our study. Additionally, our *in vitro* DOT1L enzyme activity assay revealed that API does not directly inhibit DOT1L catalytic activity, suggesting that API likely suppresses DOT1L expression or promotes its degradation rather than acting as a direct enzymatic inhibitor.

In conclusion, this study demonstrates that API inhibits the formation of senescent macrophages through hindering their phenotype conversion into the SASP. This reduction of the macrophage SASP is also revealed to be achieved through the inhibition of DOT1L-mediated H3K79me2 enrichment-induced *IL1A* expression by API. This study associated the DoT1L/H3K79me2 mechanism with aging-related chronic inflammation and confirmed that apigenin exerts its therapeutic effects by targeting this pathway. This provides a new direction for the development of selective epigenetic drugs for aging diseases. While these mechanistic insights suggest potential relevance to age-related inflammatory diseases such as atherosclerosis, further *in vivo* validation is required to confirm their therapeutic implications. Moreover, it is important to note that our study utilized THP-1-derived macrophages, a model that balances experimental tractability with biological relevance. We acknowledge that PMA differentiation may induce a pre-activated state that could influence baseline inflammatory gene expression. To mitigate this, we included a 24 h resting period after PMA removal. Nevertheless, future validation using primary human monocyte-derived macrophages or primary murine macrophages will be essential to confirm the effects of API. Furthermore, while our *in vitro* findings reveal a novel epigenetic mechanism by which API suppresses macrophage SASP, the complex multicellular environment of atherosclerotic plaques cannot be fully recapitulated in cell culture. Therefore, further studies should use an atherosclerosis model (e.g., ApoE^−/−^ mice fed a high-fat diet) to verify whether apigenin reduces macrophage senescence and the SASP in the vascular wall, and whether the DOT1L/H3K79me2-*IL1A* axis functions *in vivo*.

## Data Availability

The raw data supporting the conclusions of this article will be made available by the authors, without undue reservation.
