# Supplementary figures and images for: Targeting senescence-associated secretory phenotype macrophage: apigenin inhibits DOT1L-dependent H3K79me2 at IL1A to fight SASP in senescent macrophages
Source: Front Mol Biosci. 2026 Jul 16;13:1801780. doi: 10.3389/fmolb.2026.1801780 (PMC13421434; doi:10.3389/fmolb.2026.1801780)

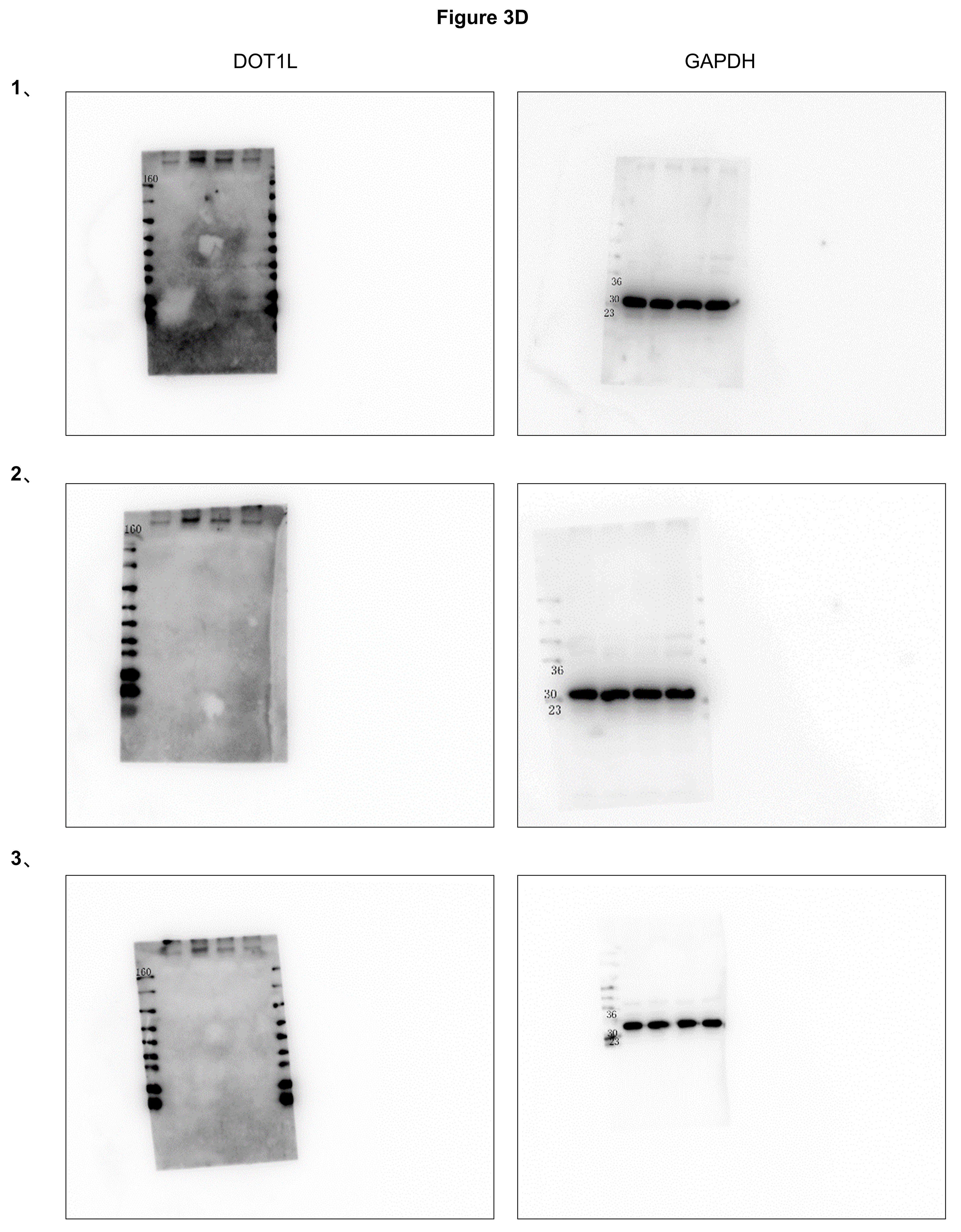

Supplement: Supplementary file 1 [file Image3.tif]

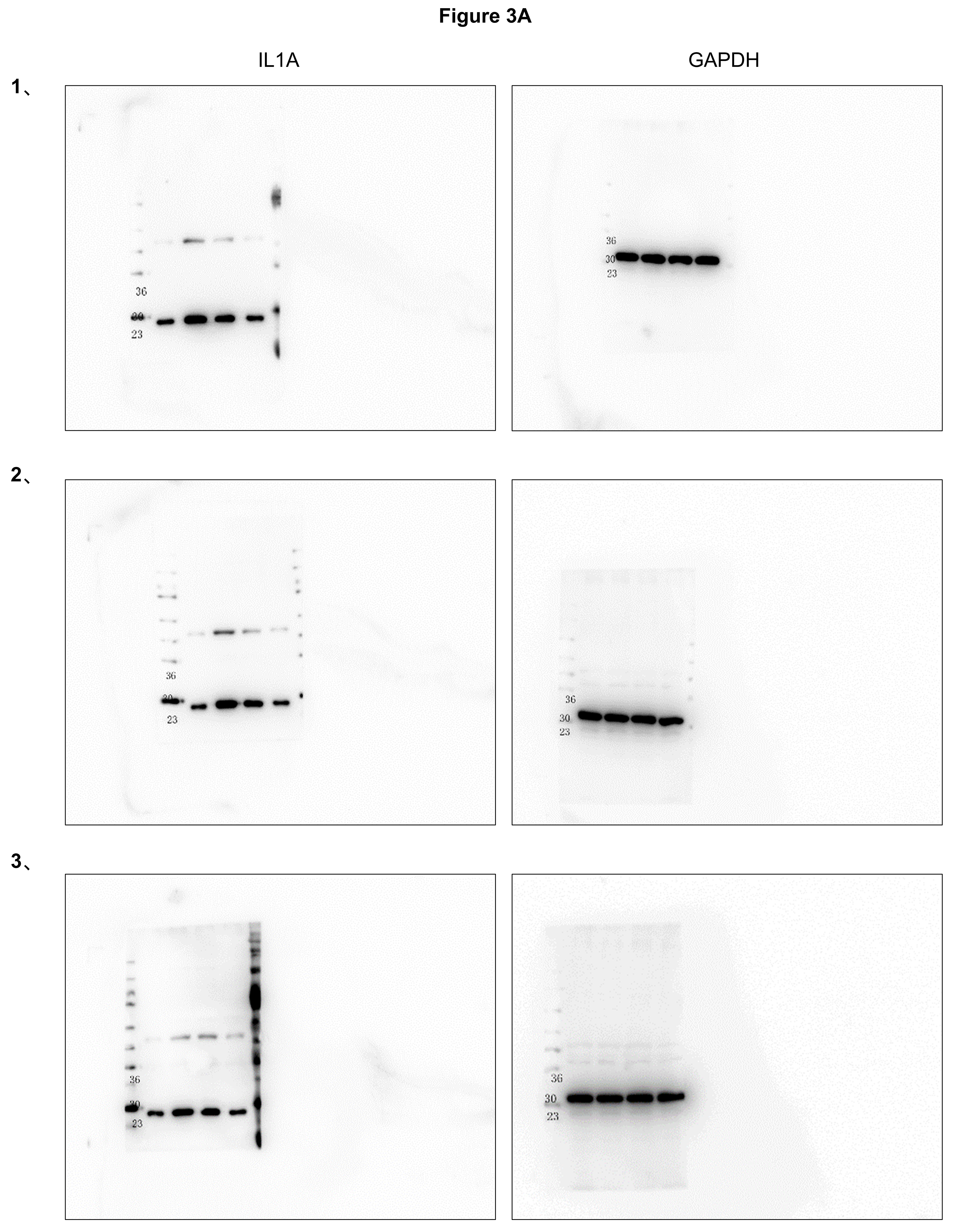

Supplement: Supplementary file 2 [file Image2.tif]

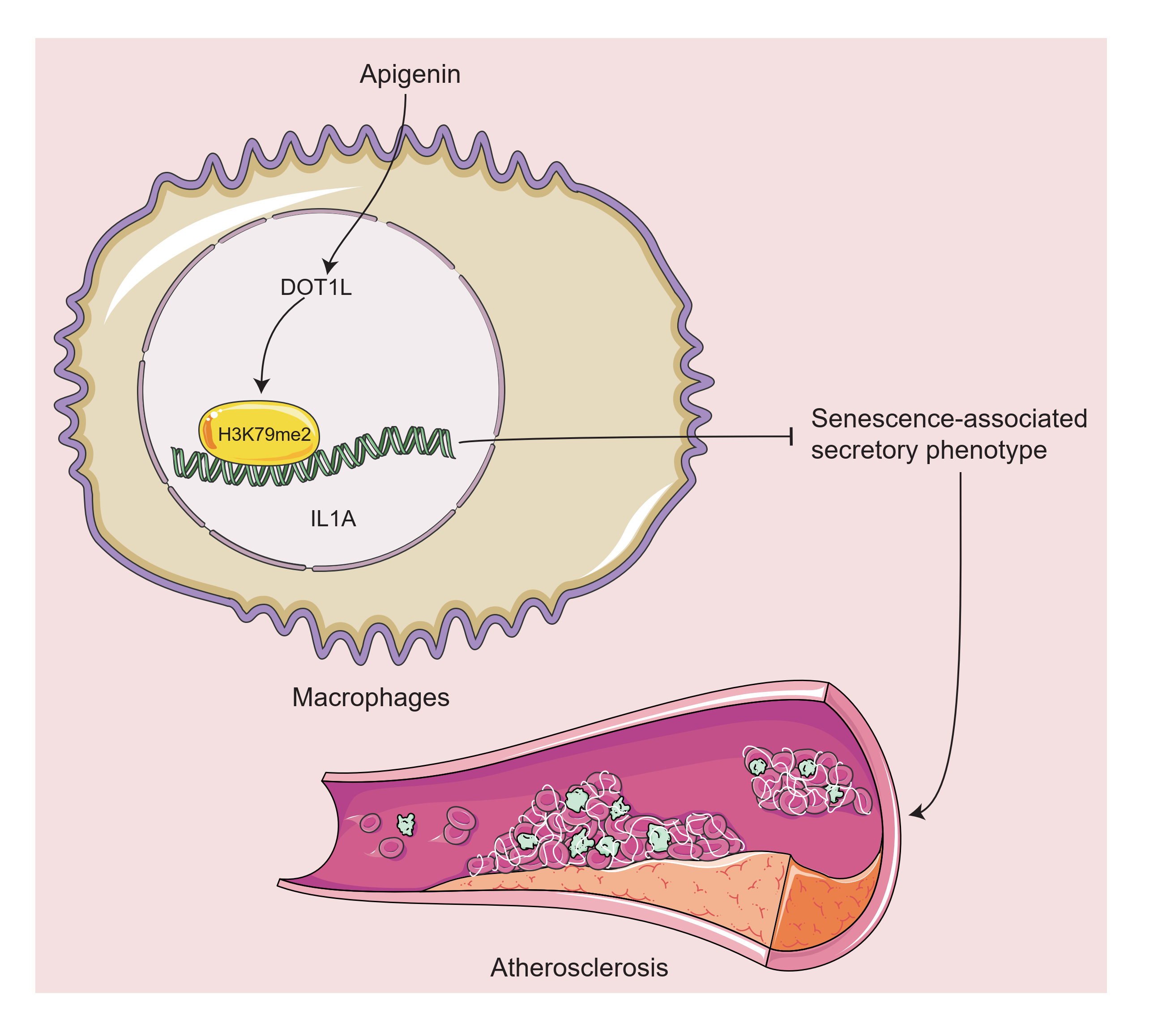

Supplement: Supplementary file 3 [file Image1.tif]
